# A Call for Leadership and Management Competency Development for Directors of Medical Services—Evidence from the Chinese Public Hospital System

**DOI:** 10.3390/ijerph17186913

**Published:** 2020-09-22

**Authors:** Zhanming Liang, Peter Howard, Jian Wang, Min Xu

**Affiliations:** 1 School of Psychology and Public Health, La Trobe University, Bundoora, VIC 3086, Australia; pfh.php@bigpond.net.au; 2 Centre for Health Management and Policy Research, School of Public Health, Cheeloo College of Medicine, Shandong University, Jinan 250012, China; jianw@sdu.edu.cn; 3 Executive Office, The Second Affiliated Hospital of Shandong First Medical University, Jinan 250033, China; x_min_1970@163.com

**Keywords:** medical directors, health service management, management workforce development, management competency, Chinese hospitals

## Abstract

*Background:* A competent medical leadership and management workforce is key to the effectiveness and efficiency of health service provision and to leading and managing the health system reform agenda in China. However, the traditional recruitment and promotion approach of relying on clinical performance and seniority provides limited incentive for competency development and improvement. *Methods:* A three-component survey including the use of a validated management competency assessment tool was conducted with Directors of Medical Services (*n* = 143) and Deputy Directors of Medical Services (*n* = 152) from three categories of hospital in Jinan, Shandong Province, China. *Results:* The survey identified the inadequacy of formal and informal management training received by hospital medical leaders before commencing their management positions and confirms that the low self-perceived competency level across two medical management level and three hospitals was beyond acceptable. The study also indicates that the informal and formal education provided to Chinese medical leaders have not been effective in developing the required management competencies. *Conclusions:* The study suggests two system level approaches (health and higher education systems) and one organization level approach to formulate overall medical leadership and management workforce development strategies to encourages continuous management competency development and self-improvement among clinical leaders in China.

## 1. Introduction

### 1.1. Development of Clinical Managers—The Pathway

Healthcare systems are unique, complex and politically sensitive, not only because of their size, but because their outputs impact directly and indirectly on the health and wellbeing of the populations that they serve. Healthcare systems require management personnel who not only have the generic management competencies, but also have a good understanding of how such complex systems function, the context in which they operate, and how the large number of organizations and sectors interact. Further limitations include dealing with constant financial constraints and the pressures of the growing healthcare needs of the population. This no doubt leads to why the increasing importance of the role of clinicians as leaders and managers and the concepts of ‘clinical leadership’ and ‘clinician turned manager’ have been well recognized [1,2]. 

For decades, the utilization of doctors in management roles has been common practice globally [3,4]. In more advanced and well-developed systems, healthcare is generally provided using the ‘clinical directorate’ concept—healthcare organizations and service provisions are managed by both clinical leaders (such as clinical directors who provide clinical leadership and manage direct service provisions) and by managing directors who may not have clinical backgrounds or qualifications and are responsible for the business and operational aspects. Very often, clinical directors carry the dual roles of heading a clinical specialty, performing management activities and maintaining their own clinical practice [4]. In a medically dominated healthcare system, clinical directors have primary control of medical practices, determine the structure and arrangements of care delivery, and manage the entire system [5]. However, this may not be the case in the less well developed and medically dominated system in China [6].

In the face of global financial downturns, a shrinking resource base and increases in demand, changing a fragmented care provision model into an integrated care model by involving clinicians, especially doctors, to manage and lead such processes, should result in the improvement of service quality, effectiveness and efficiency [2]. This makes the recruitment, selection and preparation of clinicians before and during taking on such challenging clinical management roles more critical than ever. In addition to the traditional approach of in-service training, a much more formal, systematic approach to develop clinical leaders has been recognized for decades [7].

### 1.2. Overall Health Management Workforce Development

Studies conducted in different industries and healthcare contexts over the past 20 years suggest that management competence can be acquired and improved through targeted training programs and continuous professional development [8,9,10,11]. For example, a recent study using the UK Health and Safety Executive Management Competency Framework to train financial managers in Japan demonstrated the effectiveness of training programs in developing management competence and facilitating better work engagement between managers and subordinates [10]. A recent nursing internship program implemented at the Centre for Addiction and Mental Health (CAMH) in Toronto, Canada confirmed the success of professional development and mentoring in developing nursing leaders’ competency guided by the Canadian College of Health Leaders Framework (LEADs) (2013) [11]. A randomized control trial which tested the benefits of training programs to develop the competency of public health nurses in program planning also supported the positive linkage between management training and competency development and performance improvement [12]. However, as only a small proportion of managers have the opportunity of formal management training as a mean to advancing their management careers, even in a well-developed health system such as Australia [13], informal training and development become critical. Hence, the development of a health service management workforce requires a combination of formal education such as university degree programs focusing on health service management/administration, informal training and development with more short-term and flexible approaches to management workforce development, and in-service training, mentoring and coaching [6,9]. Participation in networking activities, seminars and conferences is also a widespread approach for professional development and skill enhancement [14]. Evidence also points at the importance of using innovative pedagogy to allow integration of competencies into practice in addition to self-reflection and improvement, such as a combined approach of briefing, discussion facilitation and virtual simulation [15,16].

In Australia, Canada, the U.S., U.K. and other European countries, clinicians can formally develop their management competency via the completion of postgraduate qualifications in health administration or health service management. For example, there are 13 Master of Health Administration Programs (MHA) being offered in Australia which are accredited by the Australasian College of Health Service Management (https://www.achsm.org.au/). In the U.S. and Canada, 88 programs are accredited to the Association of University Programs in Health Administration (https://www.aupha.org/home). However, the proportion of clinicians trained by the above formal programs remains limited. There are also several specific training programs offered by medical professional institutions to foster development of doctors in leadership and management, such as the Royal Australian College of Medical Administrators (https://racma.edu.au/); the Faculty of Leadership and Medical Management in the U.K. (https://www.fmlm.ac.uk/) and the American Association of Physician Leadership in the U.S. (https://www.physicianleaders.org/).

It is argued that a cultural change in medical education is important, not only for developing medical students’ clinical skills, but also introducing some leadership and management topics into the medical curriculum to develop future clinical leaders’ understanding of healthcare policy, related issues and funding and financial arrangements [17]. The importance of leadership and management training needs to be recognized as there are core management knowledge and skills that cannot be leant solely based on experience [18].

Despite the increasing recognition of the importance of health service management, the implementation of consistent and large-scale health service management workforce development strategies can be challenging to achieve. For example, unlike other health professions in Australia health service management is not regulated by an accreditation board, resulting in no requirements for management qualifications. In addition, the management competency requirements have not been embedded in regular management performance appraisals resulting in inadequate incentives for continuous informal management training and development which are both time and financially consuming [18]. The lack of understanding of management competency requirements and competency development needs of health service managers in developing countries further limits the capacity of health service management workforce development, in particular leadership amongst clinical managers [19].

The international literature confirms the existence of core competency requirements across management levels and positions allowing learning and borrowing from competencies between different healthcare contexts [19,20]. However, the context sensitive nature of management competencies indicates that the importance of and required level of demonstration for core management competencies may vary between sectors, management positions and management levels [21]. An understanding of the extent of these differences will provide evidence to shape the design of management training and development for health service managers in specific healthcare contexts and positions [22].

### 1.3. Chinese Public Hospitals at a Glance—The Challenges and Management

The population of China slightly exceeded 1.440 billion in July 2020 equivalent to 18.5% of the total world population and the most populated country in the world [23]. Among the total of 34,000 hospitals in China, 12,000 are public and 22,000 are private. However, 85% of the 6.97 million hospital beds are located in public hospitals responsible for 85% of the 8.52 billion total hospital inpatient and outpatient consultations, [24] making the public hospital system the major medical service provider in China (another 1.89 million beds are in township healthcare centers).

As of 2019, the Chinese healthcare system employs 10.10 million medical technical personnel including 3.82 million licensed doctors and licensed assistant doctors and 4.43 million registered nurses, with majority of them currently working in the public hospital system. Approximately 4.3% of these personnel are also classified as a manager and/or have taken on dual clinician and management roles [25]. The health management workforce consisting of about half million managers is crucial to leading and supervising the transformation of the current Chinese healthcare system. This planned transformation is focused on improving the quality and cost-efficiency of health service provision by shifting from a hospital-centered and fragmentation of health service delivery approach into a more primary care-centered and integrated delivery model [25,26].

The governmental agenda of developing and expanding the healthcare landscape and the rapid development in health service provision requires a health workforce of an appropriate size, skill-mix and competency levels. Recommendations for improving the competencies for hospital managers were made in the Healthy China 2030 Program Outline and *The Guidelines Opinion of Building Modern Hospital Management Systems* published by the Chinese State Council [27]. These two governmental policy documents highlighted the important role of hospital managers in the area of hospital and medical service capacity development and the expectations of improving their professionalism and managerial skills, and the management methods/tools that they used [27]. However, as argued by Linnander et al. (2017) when comparing the health service management workforce development strategies between the USA and Ethiopia, a national framework and pathway to developing the overall health service management workforce is required [28].

### 1.4. The Recruitment and Development of Clinical Leadership and Management in the Chinese Hospital System

Similar to many developing countries, the Chinese public hospital system is still medically dominated with the vast majority of the senior hospital management positions being filled by clinicians [29,30]. A study focused on understanding the competency training needs of health executives was recently completed in three hospitals representing three different hospital categories completed in Jinan, the 19th most populated city in China with more than 4.3 million population. The study confirmed that 65% of all hospital executives are clinical directors with a medical degree with a further 28% of hospital executives (mainly Directors of Nursing) with nursing qualifications. Less than 6% of all hospital executives (mainly Directors of Administration) came from neither medical nor nursing backgrounds [6].

The senior executive positions in Chinese public hospitals, typically, Executive Directors and Deputy Directors and the Chair and Deputy Chair of the Communist Party, are appointed directly by the Provincial Health Department. The senior management positions under this executive level such as Director of Administration, Director of Clinical Services and Director of Nursing are usually selected internally based on seniority and clinical performance without specific management skills or systematic training requirements [6,29,30]. The development of managerial competency is based on experience rather than targeted management training and development [30].

Although the National Health Commission requires all health services managers to receive management training, meeting such requirements has proven challenging. Liang et al. (2020a) summed up these challenges as the following: lack of agreed management standards and requirements; irrelevance of postgraduate training in management competency development; the absence of requirement of management qualifications for management positions, and the inability of embedding the assessment of management competence and management outcomes in regular performance appraisal of hospital managers providing limited incentives for continuous management training and development [6].

In this context, a large-scale survey was conducted in three hospitals from three hospital categories in Jinan, the capital city of Shandong Province located in the northern part of China in early 2019. The study aimed to develop an understanding of hospital medical directors in terms of their education background, training received prior to and after taking up their management positions, perceived importance of management competencies to management roles, the difficulties encountered and the perceived level of management competency. The study also examined factors that may impact on the management competency development of Directors and Deputy Medical Directors. Based on the findings, the paper will discuss the proposed direction and implications for developing the health management workforce in particular the senior clinical leadership in Chinese hospitals.

## 2. Materials and Methods

A cross-sectional, descriptive study was conducted to answer the above research questions. 

### 2.1. Target Population 

The target population were Directors of Medical Services (DoMS) and Deputy Directors of Medical Services (DDoMS) working at three hospitals representing the three-tier system of hospital categorization in Jinan, Shandong, China. They were: (i) a Level 3 hospital, the First Affiliated Hospital of Shandong First Medical University, formally named Qianfoshan Hospital (QFSH), located in Jinan, the capital city of Shandong Province; (2) Lai Wu Hospital (LWH), a Level 2 hospital located in a suburb of Jinan, and (3) Xi Xian Hospital (XXH), a Level 1 hospital, located in a county area in Shandong Province. A Level 1 hospital is the equivalent to a secondary care facility based outside urban areas. Level II hospitals are equivalent to secondary care facilities based in urban areas. Level 3 hospitals are tertiary care facilities usually based in a large metropolitan center [6].

### 2.2. Questionnaire

The survey was conducted with potential participants in the targeted positions from three hospitals in Jinan City in late 2018 and early 2019. The questionnaire was developed in English and went through translation and back translation processes and pilot tested in another Jinan hospital before the Chinese version (in Mandarin) was finalized. Each questionnaire took approximately 25 min to complete and consisted of four components:

1. An explanation of the purpose of the study, instructions and consent to participate with assurance of identity protection;
2. Demography, educational background *(the lowest education category, ‘Technical College’ refers to a post school study program, a qualification or degree below that of an undergraduate or bachelor’s level.)*, and previous and current work experience;
3. Past and current management related training and management difficulties encountered;
4. Perceived importance and self-assessment of six core management competencies using the validated MCAP management competency tool, [20,31] which were:
  C1. Evidence-informed decision-making (Evidence)—13 behavioral items
  C2. Operations, administration and resource management (Resources)—17 behavioral items
  C3. Demonstrated knowledge of healthcare environment and the organization (Knowledge)—11 behavioral items
  C4. Interpersonal, communication qualities and relationship management (Communications)—19 behavioral items
  C5. Leading people and organizations (Leadership)—13 behavioral items
  C6. Enabling and managing change (Change)—9 behavioral items

The validated MCAP 7-point descriptive scale (Appendix A Table A1) was used for participants to assess their own competency levels [31]. Participants were also asked to self-assess their level of competence for the 82 behavioral items for the six competencies. The results of the self-assessments of the behavioral items associated with the six competencies will be the topic of another paper.

The Qualtrics survey platform (https://www.qualtrics.com/) was used to host the online questionnaire which was distributed by one of the QFSH Deputy Executive Directors directly to the targeted management positions at each of the three hospitals and was open for a two-week period in November and December, 2018. Three reminders were sent from the Deputy Executive Directors to all potential participants during this two-week period. Due to low response rates at QFSH, after discussions a paper-based survey with the same content as the online version was distributed in February 2019 to potential participants to encourage a higher response rate. Completed paper-based surveys were collected within two weeks.

### 2.3. Data Management and Analysis

The data were downloaded from the Qualtrics website into MS Excel format. In addition, the data from the paper-based questionnaires were entered into MS Excel. The two datasets were merged. Following error checking, the means of the six competencies and the combined competencies were calculated. All data were then imported into IBM SPSS Statistics version 25.0 (IBM Corp., Armonk, NY, USA) for analysis.

For ease of analysis, three summary scores were calculated. The first was a summary of the number of different topics of management training experienced before participants took up their management roles. The second score summarized the number of management topics taken up by participants during their management positions. The third score enumerated the number of difficulties that the participants experienced in their current position.

Univariate analyses, including tests for normality, were carried out for all variables and separately by hospital and management level. Differences between management levels and/or hospital were tested for statistical significance by crosstabulation comparing column proportions with adjusted *p-*values (Bonferroni method) and chi square tests (exact tests where indicated) or for other continuous variables by t-tests or univariate analyses of variance.

### 2.4. Ethical Approval and Consent to Participate

Ethics Approval was granted by the University Human Ethics Committee, La Trobe University (Application ID: HEC18071). All participants consented to participate in the study. This was achieved in the introductory pages of the online survey.

## 3. Results

A total of 295 DoMS/DDoMS out of a target population of 303 (97%) participated in the survey from the three targeted hospitals. Table 1 provides the characteristics of the participants by hospital. The distribution of management levels was significantly different across hospital levels. DoMS: Level 1 hospital 68.2% versus Level 3 hospital 42.6% (Chi square = 11.009, df = 2, *p* = 0.004).

### 3.1. Demography and Employment Details

The average male and female gender ratio was 1.78:1 ranging from 1.47:1 to 3.0:1 across hospitals and management levels. Overall, the mean age of participants was 47.2 years. DoMS were significantly older than DDoMS (49.4 years versus 45.1 years, t = 5.883, df = 293, *p* < 0.0005). DoMS had been employed at their current hospital significantly longer than DDoMS (26.5 years versus 20.9 years, t = 5.367, df = 293, *p* < 0.0005). DoMS had been employed as a manager significantly longer than DDoMS (12.7 years versus 7.2 years, t = 6.591, df = 293, *p* < 0.0005). DoMS had been employed in their current management role significantly longer than DDoMS (8.6 years versus 5.0 years, t = 5.893, df = 293, *p* < 0.0005).

### 3.2. Qualifications and Disciplines

Table 1 also shows the highest levels of education by hospital level. The most frequent highest level was a doctorate (*n* = 115, 39%), followed by a bachelor’s degree (*n* = 102, 35%). The next highest level was a master’s degree (*n* = 54, 18%). Finally, 8% of participants had only achieved a technical college education. The participants of this last category were usually older DoMS at the Level 1 hospital. Amongst the 169 directors with postgraduate qualifications, five of them were from LCQH (Level 2 hospital), the rest (164, 97%) worked at QFSH (Level 3 hospital). The distributions of education levels were significantly different between hospitals (Fisher’s Exact Test = 166.291 *p* < 0.0001).

There were also significant differences in the distribution of education levels between management levels (data not shown). Deputy directors had significantly higher levels of education compared to directors (Chi-Square = 21.632, df = 3, *p* < 0.0001). More deputy directors of medical services had completed a doctorate compared to directors (48.0% versus 29.4%). Moreover, directors had a higher proportion of technical college education compared to deputy directors (13.3% versus 2.6%).

Out of the 295 participants, 245 (83.1%) had degrees in medicine. Seven participants (2.4%) held a degree in nursing; 13 (4.4%) held a degree in management and 30 (10.2%) held a degree in another discipline. Only nine of the 169 postgraduate qualifications (≈5%) were management related.

### 3.3. Informal Training

Hospital managers had opportunities to participate in different types of informal training which may include management or non-management related training organized internally by the hospitals or externally by other organizations. Table 2 indicates that more managers participated in management training organized internally than externally (72% vs. 41%) for more than 10 h annually. There were no significant differences between management levels.

About 22% of participants committed to no less than ten hours in self-study of management related topics annually in the past three years. Table 2 indicates the self-study commitment by DoMS and DDoMS by hospital. Significantly more directors (65.6%) completed self-study compared to deputy directors (34.4%) (Chi square = 8.417, df = 1, *p* = 0.004). 

### 3.4. Informal Management Related Training

Overall, between 37% and 54% of managers from the three hospitals participated in some form of management related training before taking up their current management positions and between 51% and 77% of the managers from the three hospitals participated in some form of management related training after taking up their current management positions. There was an increase in management training participation amongst managers after taking up current management positions across the three hospitals. The participation rate increased between 13% and 29% (Table 3); the increase being greatest amongst managers from XXH.

### 3.5. Participation in Training Focusing on Different Management Related Topics

Sixteen management related training topics were provided to participants for multiple selection. Table 3 details the mean scores for training types undertaken before and during current management role by hospital. Managers at QFSH attended significantly more management training types both before taking up and during their management positions compared to the other two hospitals but the differences were not statistically significant. Across all hospitals, managers completed more training types after taking up the management roles compared with before. The increases were greater amongst managers at QFSH and XXH than managers at LWH but the differences were not statistically significant.

Of all the management training topics, (1) conflict resolution, (2) employee relationships, (3) safety training, (4) performance management, (5) leadership, (6) human resource management, and (7) communications were the seven areas which attracted the highest participation (26–37%) before taking up the management positions. After taking up their management roles, an additional five topics (time management, decision-making, resource management, quality control and policy and procedure) also attracted higher participation rates (27–35%).

### 3.6. Difficulties Encountered in the Management Position

**Figure 1:**
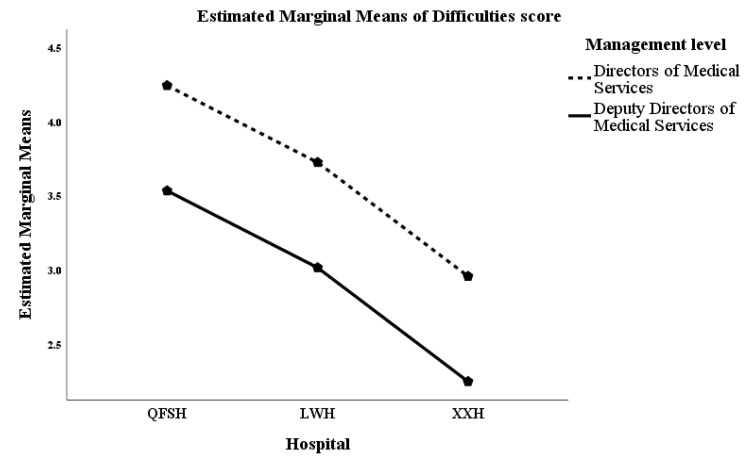
Marginal means of difficulties scores by hospital and management level. Level 1—Xi Xian Hospital (XXH); Level 2—Lai Wu Hospital (LWH); Level 3—Qian FoShan Hospital (QFSH).

A list of 15 difficulties for multiple selection were provided for participants to indicate those that they had encountered while in their current management position. There was considerable variation between hospitals. Participants at QFSH tended to report more difficulties than the other hospitals. Table 4 shows the mean difficulties scores by hospital and management level. The mean scores of QFSH managers were significantly higher than the managers at XXH (3.85 versus 2.74). The mean scores of directors of medical services were higher than those of deputy directors (3.89 versus 3.36). In a univariate model of difficulty scores both hospital and management level were significant predictors (hospital: Type III Sum of Squares = 59.206, df = 2, Mean Square = 29.603, *p* = 0.013); management level: Type III Sum of Squares = 34., df = 1, Mean Square = 34.391, *p* = 0.024). Figure 1 illustrates these results.

Table 4 also shows the percentage of managers selecting specific difficulties by management level and hospital. Patient conflict and employee performance were the difficulties selected by no less than 25% of directors from all three hospitals (bolded). Other difficulties that were selected by no less than 25% of directors from at least two hospitals included peer conflict, decision-making and change, new skill acquisition, expected work quality and management outcomes expectations (italicized). In addition, more than 25% of directors from QFSH also encountered the difficulties of team conflict, innovative teamwork and team skill building. There were few significant differences in the selection of difficulties by management level. Those selected by more than 25% of directors and deputy directors (bolded and italicized) included: peer conflict, patient conflict, innovative teamwork, employee performance, decision making and change, new skill acquisition and expected work quality.

### 3.7. Perceived Importance and Self-Assessment of Management Competencies

Participants were asked to indicate the importance of each of the six core management competencies to their current management role. Using a 5-point Likert importance scale, the vast majority of the managers (ranging from 88% to 98%) confirmed the six competencies as important or very important. 

Participants were also asked to what extent they had acquired these competencies prior to taking up the current management position using another 5-point Likert scale as detailed in Table 5. The ‘cumulative percentage’ column indicates the percentage of participants (ranging between 14.6% and 38.1%) who had not fully acquired or acquired most of the competency.

### 3.8. Overall Competency Level—Self-Assessment

Participants were asked to rate their own competency level of the six ‘overall’ competencies using the validated MCAP management competency assessment descriptive scale [25]. According to the description of MCAP Likert scale (Appendix A Table A1), a competency score of five (5.0) or greater indicates that participants could demonstrate the competency in their role independently without guidance. Table 6 provides details of the mean scores for the six competencies and the combined competencies by management level and hospital. None of the competencies received a mean score greater than five for both management levels. DoMS (range 4.33 to 4.84) scored themselves higher than DDoMS (range 3.94 to 4.57), the differences being statistically significant for competencies 2 (t = 2.350, *n* = 279, *p* = 0.019), 3 (t = 2.089, *n* = 279, *p* = 0.038), 6 (t = 2.126, *n* = 279, *p* = 0.034) and combined competencies (t = 2.128, *n* = 279, *p* = 0.034).

When analysed by hospital, managers from QFSH consistently scored themselves higher than managers from the other two hospitals. The results of analyses of variance (data not shown) showed these differences were statistically significant.

**Figure 2:**
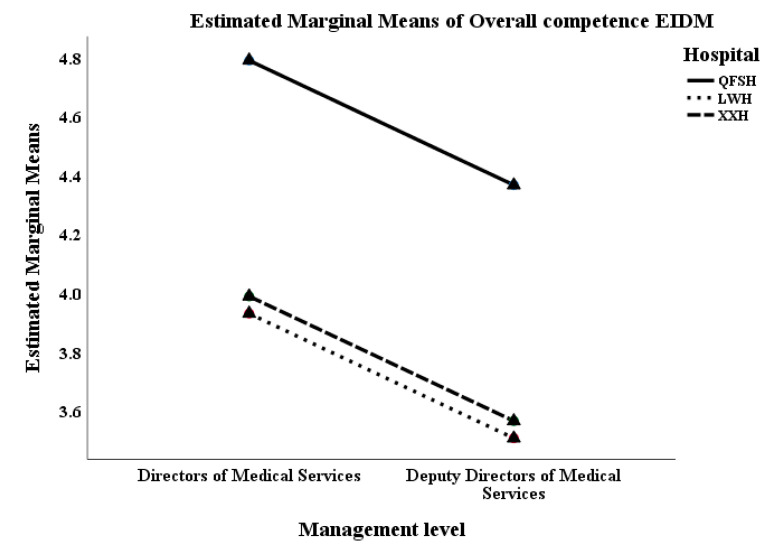
Marginal means for competency 1 (EIDM) by management level and hospital. Level 1—Xi Xian Hospital (XXH); Level 2—Lai Wu Hospital (LWH); Level 3—Qian FoShan Hospital (QFSH).

If the hospital and management level variables were included as predictors in a univariate analysis of variance model, there were significant differences between hospitals (mean square = 19.492; F = 9.649; *p* < 0.0001) and between management levels (mean square = 12.; F = 5.997; *p* = 0.015). Figure 2 is typical of all the competencies. Figure 2 demonstrates with results.

Other statistically significant predictors of the self-assessed competency levels in a bivariate relationship included age (positive correlation), total number of years as a manager (positive correlation). hospital, management level and qualifications (undergraduate > postgraduate). Including these predictor variables into a univariate model with an interaction term for age/total years as a manager (highly correlated), age was the only consistently significant predictor of the scores of all six competencies and the combined competencies scores. Total years as a manager was also a significant independent predictor for competencies 4 (communications) and 5 (leadership). None of the interaction terms were significant.

## 4. Discussion

The survey achieved a 97% response rate demonstrating the support and commitment from the participating hospitals and the perceived importance and relevance of the study. The findings of the survey confirm the importance and timeliness of the study. As discussed earlier, the rapidly changing healthcare landscape and the pressure of transformation of the Chinese hospital system signal the demand for a highly skilled and resilient health service management workforce [6]. As Chinese public hospitals provide more than 80% of medical services across the country and have been medically dominated since their establishment, the development of clinical leaders in particularly medical directors is essential [25]. Current medical curricula are focused primarily on the development of clinical skills and medial expertise with no coverage of leadership and management competencies. The tradition of ‘clinician turned manager’ continues to be determined by seniority and clinical performance. Consequently, the development of a competent management workforce in Chinese public hospitals is challenging and requires a framework of guidance with a more holistic and systematic approach. 

This study suggests that a review of the requirements for qualifications and participation in informal training among clinical leaders (DoMS and DDoMS) is indicated, particularly in the better resourced and more competitive Level 3 hospital. This maybe a reflection of the recognition of the importance of a competent health service management workforce and its development needs at the central government level, clearly emphasized by the ’Healthy China 2030 Program Outline’ and *The Guidelines Opinion of Building Modern Hospital Management Systems* [32]. The study found that about half of the deputy directors of medical services in QFSH (Level 3 hospital) possessed doctorate level qualifications which was significantly higher than the directors in the same hospital and among colleagues at the same management level at the other two hospitals. Explanations would include the commitment of Level 3 hospitals to a greater research responsibility and the more competitive nature of a younger generation of medical directors.

Although higher qualifications (master’s and doctorates) were possessed by a much larger proportion of the younger generation of directors (deputy directors were on average four years younger than directors), less than six percent of these degrees were management related which may explain the findings of the study—possession of higher qualification was not positively associated with an increase self-assessed confidence in management competency. On the other hand, given significantly higher proportion of directors of medical services had committed to self-study in management related topics than deputy directors and had gained average six to eight years of additional management experience, the positive correlation between age and self-assessed management competency levels is not surprising. 

Whilst formal higher education was not focused on improving management competency, informal training in management related topics, self-study and wisdom gained from actual management experience become important. However, this cannot relegate the importance of formal education and training in health service management as the finding of self-assessed management competency scores of less than five amongst both management levels across three different levels of hospital is of concern (a score of five is the distinction between competent requiring guidance and competent without guidance).

### 4.1. Lack of Self-Assessed Management Competence

Despite the recognition of the importance of the six core management competencies for management roles, not all medical directors felt that they had fully acquired or acquired most of the competencies before taking up medical directorship, with a higher proportion (more than 30%) for competency 2 (Resources), competency 5 (Leadership) and competency 6 (Change). This may explain why they gave themselves an overall score less than five for each of the six management competencies ranging between 4.31 and 4.89 with a combined six competencies score of 4.61. Consistently, although not statistically significant, C6 (Change) received the lowest score among all six competencies across management levels and hospitals, followed by C2 (Resources). More alarming, deputy directors of medical services and medical directors from LWH and XXH scored less than four for C6 (Change) indicating a self-perception of not being fully competent in demonstrating the competency in their management role. Such low levels of self-assessed competence was not identified in similar studies in Australia targeting senior and middle level managers [13,20].

This further confirms that the possession of postgraduate qualifications (more than 57% of all medical directors possessed postgraduate qualifications with vast majority of these possessed by deputy directors’ medical directors from QFSH) and a higher level of participation in management related training before and after taking up the management positions (50–66%) are not linked to satisfactory scores of self-assessed management competence. A possible conclusion is that the management related training undertaken was less than effective.

Medical directors are the highest level of clinical leaders in Chinese Public hospitals who hold the responsibility for clinical service provision and resource allocation and influence the quality and safety of patient care and are also central to the complex patient–doctor relationship and disputes [6,33]. Medical directors should also play a key role in providing leadership, mentoring and coaching to junior level managers as future clinical leaders. Their low level of commitment to self-study (only 22% all medical directors committed to more than 10 h annually) and informal training, accompanied by their low self-assessed competency scores raises questions of how to develop and sustain a competent health service management workforce in China to meet the increasing healthcare demands in public hospitals and manage and lead a successful health system reform agenda [25,26].

Chinese public hospitals urgently require not only effective clinical leadership with improved management competence, but also a vision for appropriate strategies that can lead to the development of a sustainable management workforce that plays an essential leading role in managing the challenges facing the Chinese public health system.

### 4.2. Training, Difficulties, Competencies and Implications

As mentioned earlier, in the medically dominated public hospital system, the recruitment of medical directors is primarily based on seniority and clinical performance providing inadequate incentives for taking up management related training [6,29,30]. Clinicians face heavy workloads and are encumbered by the financially driven public hospital funding model and more complex patient–doctor relationships [33,34,35,36]. Empirical evidence indicates that the erosion of trust in the medical profession, poor communications and attitudes including de-valuing patients’ views by medical professionals are two of major reasons behind the medical disputes in Chinese hospitals [29,30]. Highlighting that good communications and interpersonal skills are tools that help improving patient satisfaction and quality of patient care [37].

However, performance of the medical leaders is likely to be assessed by clinical performance and the ability to meet financial targets and profit benchmarks, rather than overall management outcomes such as efficiency in resource allocation and work processes, and further assessed by immediate clinical outcomes rather than long term improvement of patients’ health and wellbeing [33]. In this context, relying on self-motivation to develop and improve management competency, and the ability to use tested management tools and methods without specific formal and informal management training is a major challenge [38].

The study confirms that all medical directors across hospitals and management levels have encountered difficulties in their management positions, in particular those at the most senior level—directors of medical services and those who are working at the Level 3 hospital. These difficulties relate to:

- Dealing with conflicts with patients, staff members and their peers;
- Improving and managing performance: staff performance, service quality and management outcomes;
- Developing new skills; and
- Making decisions and managing change.

Examining the main difficulties in reference to the detailed behaviors associated with the six core management competencies, it is clear that all of the competencies are important to successfully overcome the main difficulties encountered. However, the low level of self-assessed competency is likely to be an obstacle in itself, suggesting that further formal and informal management training will be essential for not only overcoming difficulties but also for maintaining the expected clinical and management performance outcomes, confirming the importance of overall management competency development for medical directors.

Furthermore, the fact that the difficulties encountered are common across management levels and hospitals, such difficulties may not be a result of a specific local hospital context or patient cohorts, but factors that impact on the overall public hospital system and hospital management workforce. Therefore, system-wide policy development and strategies are required.

In addition, reviews of organization-based policies and strategies and of medical curricula should occur in conjunction with the policy reviews at the system level. There is also empirical evidence that has not only championed the importance of self-improvement and life-long learning in enabling work efficiency and career advancement, but also its ability to instill a sense of purpose, self-worth and self-assessed confidence [39].

To summarize the above discussion (see Table 7), the authors suggest that strategies to develop the overall leadership and management competency of clinical directors for Chinese public hospitals beyond individual levels should at least focus on three levels: (1) two system levels: health system and higher education system [6,13,14,17,18,27,28]; and (2) healthcare organization level [6,8,9,13,14,31].

The successful implementation of the above strategies would ultimately develop a culture that encourages continuous management competency development and self-improvement among clinical leaders who can lead and manage the health system reform agenda and maintain and improve the quality of health service provision which is important to a sustainable healthcare system that can meet the increasing healthcare needs the population. 

### 4.3. Strengths and Weaknesses

The major strength of the study is the sample size and high response rates across hospitals. One weakness of the study was the reliance on self-reported information which may challenge its objectivity. However, any error introduced is likely to randomly distributed, although a degree of common method bias cannot be excluded. In addition, the results are based on a study in three hospitals from one province, so its external validity may be limited nationally.

## 5. Conclusions

The study confirms that core management competencies identified in a non-Chinese context are also core to medical directors in the Chinese public hospitals. Despite the recognition of their importance, medical directors across three Chinese public hospital levels have not sufficiently acquired such competencies prior to taking up their senior medical leadership roles. The lack of effective formal and informal training in management related areas may have attributed to the low self-assessed management competency levels. The study strongly argues the importance of informal management training, coaching and mentoring for developing the medical leadership and management without downgrading the importance of formal management training.

To develop and sustain an effective medical leadership and management workforce in China, the paper champions two-system level (health system and higher education system) and one healthcare organization level approaches to formulate overall workforce development strategies. The successful implementation of such strategies would lead to the development of a culture that encourages continuous management competency development and self-improvement among clinical leaders. Investment in the capability development and competency improvement of the medical leaders in China is critical and could lead to improved quality of service provision with greater economic sustainability and improved public health outcomes.

## Figures and Tables

**Table 1:** Characteristics of participants by hospital.

Position		Hospital Level			
		Level 1 *	Level 2 *	Level 3 *	Total
**Directors of Medical Services**	**Count (%)**	30 (68.2) _a_	24 (57.1) _a, b_	89 (42.6) _b_	143 (48.5)
Deputy Directors of Medical Services	Count (%)	14 (31.8) _a_	18 (42.9) _a, b_	120 (57.4) _b_	152 (51.5)
Total	Count	44	42	209	295
**Sex**		**Level 1**	**Level 2**	**Level 3**	**Total**
Male	Count (%)	33 (75.) _a_	25 (59.5) _a_	131 (62.7) _a_	189 (64.1)
Female	Count (%)	11 (25.0) _a_	17 (40.5) _a_	78 (37.3) _a_	106 (35.9)
**Highest education level**		**Level 1**	**Level 2**	**Level 3**	**Total**
Technical college	Count (%)	15 (34.1) _a_	4 (9.5) _b_	4 (1.9) _c_	23 (7.8)
Bachelor’s degree	Count (%)	29 (65.9) _a_	33 (78.6) _a_	40 (19.2) _b_	102 (34.7)
Master’s degree	Count (%)	0 (0.0) _a_	5 (11.9) _a, b_	49 (23.6) _b_	54 (18.4)
Doctorate	Count (%)	0 (0.0) _a_	0 (0.0) *_a_*	115 (55.3) _b_	115 (39.1)
**Age**		**Level 1**	**Level 2**	**Level 3**	**Total**
	Count	44	42	209	295
	Median (IRQ)	41.0 (9)	46.0 (4)	48.0 (12)	47.0 (11)
**Years at current hospital**		**Level 1**	**Level 2**	**Level 3**	**Total**
	Count	44	42	209	295
	Median (IRQ)	17.50 (13)	23.00 (9)	25.00 (13)	24.00 (13)
**Years as manager**		**Level 1**	**Level 2**	**Level 3**	**Total**
	Count	44	42	209	295
	Median (IRQ)	8.0 (15)	8.0 (12)	9.0 (12)	8.00 (12)
**Years in current management position**		**Level 1**	**Level 2**	**Level 3**	**Total**
	Count	44	42	209	295
	Median (IRQ)	3.0 (6)	6.0 (9)	4.0 (8)	4.0 (8)

* Level 1—Xi Xian Hospital (XXH); Level 2—Lai Wu Hospital (LWH); Level 3—Qian FoShan Hospital (QFSH). The proportions shown are based on a comparison of columns. The compare column proportions option computes pairwise comparisons of column proportions and indicates which pairs of columns (for a given row) in the crosstabulation table are significantly different. The column proportions test assigns a subscript letter to the categories of the column variable. For each pair of columns, the column proportions (for each row) are compared using a *z test*. If a pair of values is significantly different, the values have different subscript letters assigned to them.

**Table 2:** Proportion of participants undertaking different types of informal training for more than 10 h annually by management level, and proportion undertaking self-study by management level and hospital.

	Training Type				Self-Study of Management-Related Topics			
					Hospital Level			
**Management Level**	**Internal Management**	**External Management**	**Internal Non-Management**	**External Non-Management**	Level 1 *	Level 2 *	Level 3 *	**Combined**
**Directors of Medical Services**	69.0%	40.8%	54.9%	54.2%	28.6%	7.1%	64.3%	65.6%
**Deputy Directors of Medical Services**	75.0%	40.3%	57.6%	45.1%	4.5%	13.6%	81.8%	34.4%
**All Directors**	72.0%	40.6%	56.3%	49.7%	20.3%	9.4%	70.3%	100%

* Level 1—Xi Xian Hospital (XXH); Level 2—Lai Wu Hospital (LWH); Level 3—Qian FoShan Hospital (QFSH).

**Table 3:** Frequency and proportion (*n* (%)) of participants taking part in management related training before taking up and during their current management positions by hospital, and mean scores of training types completed by hospital.

	Percentage of Participants			Mean Score of Training Types		
	Before	During	Increase	Before	During	Increase
Level 1 *	20 (48)	33 (77)	29%	2.18	3.45	58%
Level 2 *	15 (37)	21 (51)	14%	3.14	3.52	12%
Level 3 *	108 (54)	136 (67)	13%	3.28	4.44	35%
**Total**	143 (50)	190 (66)	16%	3.10	4.16	34%

* Level 1—Xi Xian Hospital (XXH); Level 2—Lai Wu Hospital (LWH); Level 3—Qian FoShan Hospital (QFSH).

**Table 4:** Mean difficulty scores and difficulties experienced (percentage of managers) by hospital and management level.

	Level 1 *	Level 2 *	Level 3 *	DoMS #	DDoMS #
**Mean Difficulty Scores**	2.74	3.43	3.85	3.89	3.36
**Difficulties**	**Level 1**	**Level 2**	**Level 3**	**DoMS (%)**	**DDoMS (%)**
Peer conflict	23	*25*	*29*	*27*	*28*
Team conflict	19	23	27	28	23
Staff turnover	21	23	5	14	5
Patient conflict	**40**	**48**	**50**	***46***	***50***
Innovative teamwork	14	23	41	***38***	***31***
Staff hiring	5	3	9	7	7
Loss of skilled staff	16	18	10	13	11
Team skill building	14	8	26	23	21
Ethical problems	2	5	11	9	11
Supervisor confrontation	12	3	9	12	5
Employee performance	**28**	**35**	**41**	***40***	***36***
Decision-making & change	*30*	18	*31*	***33***	***25***
New skill acquisition	19	*45*	*33*	***35***	***31***
Expected work quality	14	*43*	*33*	***34***	***29***
Management outcomes expectations	16	*28*	*27*	30	22

* Level 1—Xi Xian Hospital (XXH); Level 2—Lai Wu Hospital (LWH); Level 3—Qian FoShan Hospital (QFSH). # DoMS—Director of Medical Services; DDoMS—Deputy Directors of Medical Services. Bolded percentages identify difficulties experienced by more than 25% of managers from all three hospitals. Italicized percentages indicate difficulties experienced by more than 25% of managers from two hospitals. Those bolded and italicized identify difficulties experienced by more than 25% of both DoMS and DDoMS.

**Table 5:** Proportions of managers acquiring competencies before taking up their current management position.

Competency	Not at All	Acquired to Limited Degree	Unsure	Cumulative Percentage	Acquired Most of It	Fully Acquired
**C1 Evidence**	2.4%	9.1%	15.4%	26.9%	55.6%	17.5%
**C2 Resources**	5.9%	8.4%	23.8%	38.1%	49.0%	12.9%
**C3 Knowledge**	0.0%	5.2%	10.1%	15.3%	61.9%	22.7%
**C4 Communications**	0.0%	5.2%	9.4%	14.6%	59.1%	26.2%
**C5 Leadership**	3.8%	8.7%	20.3%	32.8%	50.7%	16.4%
**C6 Change**	6.3%	10.1%	21.0%	37.4%	46.5%	16.1%

**Table 6:** Mean scores of self-assessed management competencies by management level and hospital.

Competencies	Management Level		All Directors	Hospital Level		
	DoMS #	DDoMS #		Level 1 *	Level 2 *	Level 3 *
**C1. Evidence**	4.47	4.19	4.33	3.86	3.75	4.55
**C2. Resources**	4.44	4.02	4.23	3.74	3.63	4.45
**C3. Knowledge**	4.78	4.42	4.59	4.07	3.98	4.83
**C4. Communications**	4.84	4.57	4.70	4.37	4.15	4.89
**C5. Leadership**	4.56	4.23	4.40	3.86	3.88	4.62
**C6. Change**	4.33	3.94	4.14	3.84	3.58	4.31
**Six competencies**	4.57	4.23	4.40	3.96	3.83	4.61

* Level 1—Xi Xian Hospital (XXH); Level 2—Lai Wu Hospital (LWH); Level 3—Qian FoShan Hospital (QFSH). # DoMS—Director of Medical Services; DDoMS—Deputy Directors of Medical Services.

**Table 7:** Three levels of strategic development for clinical leaders and managers.

Level	Strategies
**Health system** **[6,18,27,28]**	▪ Establish national level standards for clinical leaders/managers by defining management qualifications and competency requirements. ▪ Develop a national policy that recognizes the importance of health service management positions and identify indicators to measure successful management outcomes. ▪ Incorporate continuous professional development as requirement for managers to maintain standards.
**Higher education system** **[6,14,17]**	▪ Work closely with public hospitals to identify strategies to bridge the knowledge gaps that are fundamental to further develop clinical leadership and management. ▪ Review and revise undergraduate medical curricula to introduce leadership and management related concepts that will provide an understanding of the roles of clinical leaders/managers. In addition, incorporate some leadership and management knowledge and skills into clinically based master’s degrees to prepare medical doctors to take up medical leadership and management roles. ▪ Further development of non-research-based postgraduate courses in health service management
**Healthcare organisation** **[6,8,9,13,14,31]**	▪ Develop management position job descriptions and a hierarchy for clinical leadership development. ▪ Formulate succession planning and recruitment strategies for senior management positions with clear qualification and competency requirements. ▪ Embed management competency assessment as part of the performance review process to identify competency gaps. ▪ Developing an organization-wide support and training framework, using a mixed approach including the provision of targeted training, support, mentoring and on the job coaching.

## Appendix A

**Table A1:** MCAP Competency Likert Scale for self-assessment.

Scale *	Level	Competency Level
1	Not competent	Do not understand the requirements and am not capable of applying it to my role
2	Basic or novice	May be capable of demonstrating minor aspects in my role
3	Advanced beginner	May be capable of demonstrating in my role, but not in all required aspects
4	Competent with occasional guidance	Can generally demonstrate in my role, but guidance is needed occasionally
5	Competent, no guidance	Can demonstrate in my role independently without guidance, but have not had extensive experience
6	Proficient	Always apply appropriately in my role with extensive experience
7	Superior expertise	Always apply appropriately in my role with extensive experience gained from diverse management roles at executive level and can teach this competency to others

* Scores less than five are considered less than fully competent. Scores five or greater are considered fully competent.
